# High‐Frequency Repetitive Transcranial Magnetic Stimulation on the Left Parietal Lobe Improves Post‐Stroke Memory Impairment: An fNIRS Study

**DOI:** 10.1002/brb3.71355

**Published:** 2026-03-31

**Authors:** Luhui Cai, Yuye Sun, Yijiang Li, Peirong Wu, Mindong Wei, Yinuo Bi, Chaowen Wang, Wenyu Jiang

**Affiliations:** ^1^ Department of Neurological Rehabilitation Jiangbin Hospital of Guangxi Zhuang Autonomous Region Nanning China; ^2^ Department of Rehabilitation The First People's Hospital of Yulin Yulin China; ^3^ Data Science with Artificial Intelligence University of Exeter England UK; ^4^ Cognitive Rehabilitation Center Jiangbin Hospital of Guangxi Zhuang Autonomous Region Nanning China

**Keywords:** fNIRS, high‐frequency rTMS, left parietal lobe, post‐stroke memory impairment

## Abstract

**Background:**

Memory impairment is a prevalent cognitive deficit following stroke, which can significantly impact quality of life and rehabilitation prognosis. Traditional cognitive rehabilitation therapy offers some benefits in improving memory function, its efficacy remains limited. This study investigated the effects of high‐frequency repetitive transcranial magnetic stimulation (rTMS) at the left parietal lobe on post‐stroke memory impairment.

**Methods:**

A total of 45 patients with post‐stroke memory impairment were randomly divided into two groups. The rTMS group underwent 20 Hz rTMS on the left parietal lobe, while the non‐stimulated control group received sham stimulation. All participants underwent neuropsychological tests (including the montreal cognitive assessment (MoCA), Rey auditory verbal learning test (RAVLT), and Rey‐Osterrieth complex figure test (ROCF)) and functional near‐infrared spectroscopy (fNIRS) scans before and after intervention. fNIRS data was analyzed by functional connectivity analysis to measure changes within brain networks.

**Results:**

After treatment, the rTMS group showed greater improvement in MoCA scores compared to baseline (*p* < 0.05). Relative to the controls, the rTMS group demonstrated higher scores in delayed recall for RAVLT and ROCF (*p* < 0.05), as well as significantly enhanced resting‐state functional connectivity (rsFC) within the prefrontal lobe and between the left prefrontal lobe and right occipital lobe (*p* < 0.05).

**Conclusions:**

High‐frequency rTMS of the left parietal lobe effectively ameliorates memory impairment in stroke patients, and one of the possible mechanisms is the enhanced rsFC within the prefrontal lobe and between the prefrontal and occipital lobes.

## Graphical abstract

This study investigated the effects of 20 Hz repetitive Transcranial Magnetic Stimulation at the left parietal lobe on post‐stroke memory impairment. The results showed that the memory function of stroke patients improved significantly, and the resting‐state functional connectivity within the prefrontal lobe as well as between the prefrontal and occipital lobes were significantly enhanced.

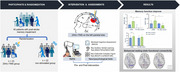

## 1 Introdution

Memory impairment is a common cognitive deficit after stroke, which can significantly impact activities of daily living, quality of life, and rehabilitation prognosis (Jokinen et al. 2015, O'Sullivan et al. 2023). Research has indicated that memory impairment might lead to a variety of emotional and behavioral abnormalities, and even further worsen the overall cognitive function (Reed et al. 2004). While traditional cognitive rehabilitation therapy offers some benefits in improving memory function, its efficacy remains limited (Piras et al. 2011; Das Nair et al. 2016).

rTMS involves delivering short, repetitive, high‐intensity magnetic field pulses to specific brain regions. Its high‐frequency pattern enhances cortical excitability, regulates neural activity, and reshapes brain functioning (Valero‐Cabré et al. 2017). rTMS has gained widespread use in clinical practice due to its precise targeting capabilities, individualized adjustments, and non‐invasive nature with safety assurance. Studies have demonstrated the positive effects of rTMS on various cognitive domains such as memory, speech, and executive function (Wang and Voss 2015; Bai et al. 2022; Gao et al. 2023), thus it can be employed in combination therapies for treating cognitive disorders. The parietal lobe, as an important constituent of the parietal memory network (PMN), supports memory encoding, integration, storage and retrieval functions through mutual connection and synergy with other brain regions in the network (Gilmore et al. 2015). Even in the absence of direct parietal damage in stroke patients, damage to other parts of the brain may lead to functional disconnection, thereby causing reduced PMN activity and resulting in memory deficits. A previous case study used high‐frequency rTMS to target the left inferior parietal lobule to treat a patient with amnestic mild cognitive impairment (Cotelli et al. 2012) and found that the memory performance improved significantly, but the neural mechanism was not deeply studied.

The fNIRS is a non‐invasive method for imaging brain function, which can directly observe the hemodynamic response of the cerebral cortex at each moment by recording the increase and decrease in oxygenated hemoglobin and deoxyhemoglobin concentration. These responses are closely coupled with neuronal activity through neurovascular coupling, which can be used to speculate the neural regulation of brain activity (Pinti et al. 2020). Compared with fMRI, PET, and other technologies, although fNIRS has relatively limited spatial resolution and penetration depth, it is relatively low‐cost, portable and easy to operate, and has fewer physical environment restrictions, which is especially suitable for monitoring in clinical conditions. When combined with clinical neuromodulation therapy technology, fNIRS serves as a powerful tool to evaluate efficacy in real‐time and offers new opportunities to explore changes in brain function. Its applications have been widely observed in research related to Alzheimer's disease, stroke, depression, and other disorders (Yeung and Lin 2021; Zhang et al. 2022; Kim et al. 2023). rsFC, defined as the correlation of physiological activities between different neural networks within the brain, represents an important index used to detect brain function. It can reflect the internal brain activity characteristics and reveal the synergistic effects among various brain regions. Zhang et al. (Zhang et al. 2011) performed a test‐retest analysis using independent component analysis to verify the reliability of fNIRS‐rsFC. Another simultaneous recording study found a direct correlation between fNIRS‐rsFC values and fMRI‐rsFC values, indicating that brain optical connectivity is related to brain functional structure (Duan et al. 2012). These prove that the fNIRS‐rsFC has good validity and reliability.

Therefore, our study focused on post‐stroke patients with memory impairment and investigated the effect of high frequency rTMS stimulation of the left parietal lobe on memory function using a randomized controlled design. Furthermore, we aimed to investigate changes in brain oxygenation and rsFC using fNIRS imaging technique to explore neuroimaging mechanisms and provide valuable insights for clinical decision‐making.

## 2 Methods

### 2.1 Participants

The study participants were recruited from inpatients at the Jiangbin Hospital of Guangxi Zhuang Autonomous Region, China. From January 2021 to June 2022, 45 patients with post‐stroke memory impairment who met the criteria were recruited. The patients were randomly divided into the rTMS group (*n* = 23) and the non‐stimulated (no‐stim) control group (*n* = 22). This study was approved by the Jiangbin Hospital of Guangxi Zhuang Autonomous Region Ethics Committee. All study participants provided informed written consent for inclusion in the research and publication.

All subjects satisfied the following criteria: (1) met the criteria for stroke diagnosis, and confirmed by head CT or MRI examination; (2) first onset, disease course 2 weeks to 6 months, right‐handed, aged 18 years or older; (3) stable vital signs, no progression of stroke symptoms; (4) subjective memory impairment; (5) MoCA score < 26, and MoCA > 5, and memory subtest indicates memory impairment; and (6) Able to cooperate with related assessments and treatment.

Subjects with any of the following conditions will be excluded: (1) serious visual and auditory impairments; (2) metal implants, pacemakers or skull defects that cannot be treated with rTMS; (3) previous history of brain tumor, head injury, epilepsy, or other conditions that cause seizures; (4) aphasia; (5) accompanied by severe comorbidities and complications; (6) cognitive impairment before stroke; and(7) unable to cooperate with clinical examination for various reasons.

Both groups were given basic treatment: (1) drug therapy such as secondary prevention of stroke, improvement of cognitive function, etc.; and (2) conventional rehabilitation training: targeted cognitive function training such as computer‐assisted cognitive function training was carried out according to the results of cognitive function assessment. The training was conducted by two certified rehabilitation trainers.

### 2.2 Randomization and Blinding

Patients were randomly assigned to two groups by an independent research assistant using a computer‐generated list of random numbers, followed by group concealment using sealed, opaque envelopes. These envelopes were opened only when participants were enrolled. Subjects, care providers, outcome observers, and rehabilitation trainers were blinded to group allocation. rTMS was performed by an independent therapist who was instructed not to discuss the intervention details with other personnel throughout the treatment. Patients and their caregivers were asked not to discuss their treatment allocation with staff or other patients.

### 2.3 Neuropsychological Measurements

All subjects underwent a formal neuropsychological assessment by a trained neuropsychological technician, and the accuracy of the results was verified by 2 professionals. Memory function was assessed using the MoCA, RAVLT, and ROCF. All the assessments were conducted before treatments and after 3‐week treatments (the day after the final treatments).

RAVLT is designed to evaluate episodic memory (Lavoie et al. 2018), encompassing RAVLT immediate recall (RAVLT‐IR), and RAVLT delayed recall (RAVLT‐DR). After conducting five consecutive learning and recall sessions for 15 nouns (Group A), learning and recall were carried out for another 15 interfering nouns (Group B), and the words of Group A are recalled 20 min later. The total score of the correct recalls in the first five sessions constitutes the immediate memory score, which is utilized for assessing immediate memory. The total score was 75 points, and a score greater than 15 was considered normal. Moreover, the total score of the correct recalls after 20 min constituted the delayed memory score, which is employed for evaluating delayed memory. A score greater than 4 was regarded as normal. Higher scores indicated greater auditory episodic memory ability.

ROCF is a commonly utilized test approach for assessing visual spatial structural function and visual memory abilities (Cherrier et al. 1999). ROCF comprises repeated squares, rectangles, triangles, and a variety of other shapes, with a total of 36 points. First, the subject is requested to copy the complex graphic within 5 min, namely ROCF copying (ROCF‐C), and is instructed to remember the graphic while copying. After the 20–30 min, the subject is requested to recall and draw the graphic, which is referred to as ROCF delayed recall (ROCF‐DR). Higher scores indicated greater visual episodic memory ability of the subject.

### 2.4 rTMS Procedure

**FIGURE 1:**
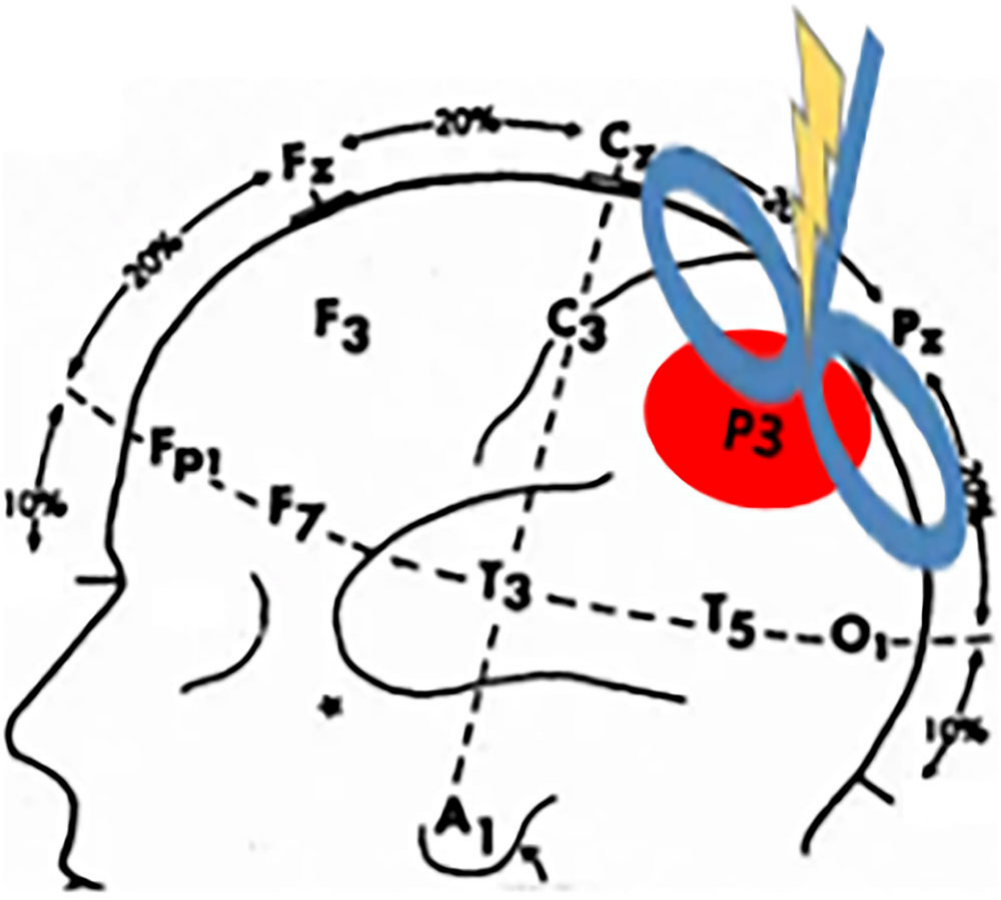
The rTMS stimulation target: the left parietal lobe (at P3 in the international 10–20 EEG system).

rTMS treatment was conducted with a CCY‐1A magnetic stimulator (The Yiruide Medical Technology Co., Ltd., Wuhan, China) and a standard figure‐of‐eight coil. The stimulated area was the left parietal lobe (at P3 in the international 10–20 EEG system, as shown in Figure 1). The center of the coil was aligned with the stimulated site and tangent to the surface of the patient's scalp. Then, 20‐Hz rTMS was applied at 80% RMT, with trains of 2‐s duration, 22‐s inter‐train interval, and a of total 2000 pulses costing 20 min on the left parietal lobe each day, 5 days per week for 3 weeks. For the no‐stim control group, the coil was placed perpendicularly to the surface of the skull, resulting in no magnetic field.

The intensity for each patient was calibrated by a professional therapist prior to each intervention for safety purposes. Throughout the treatment, close observation was conducted of the patient for symptoms such as headache, tinnitus, epilepsy, etc., and immediate cessation of treatment occurred upon detection.

### 2.5 fNIRS Data Acquisition and Preprocessing

**FIGURE 2:**
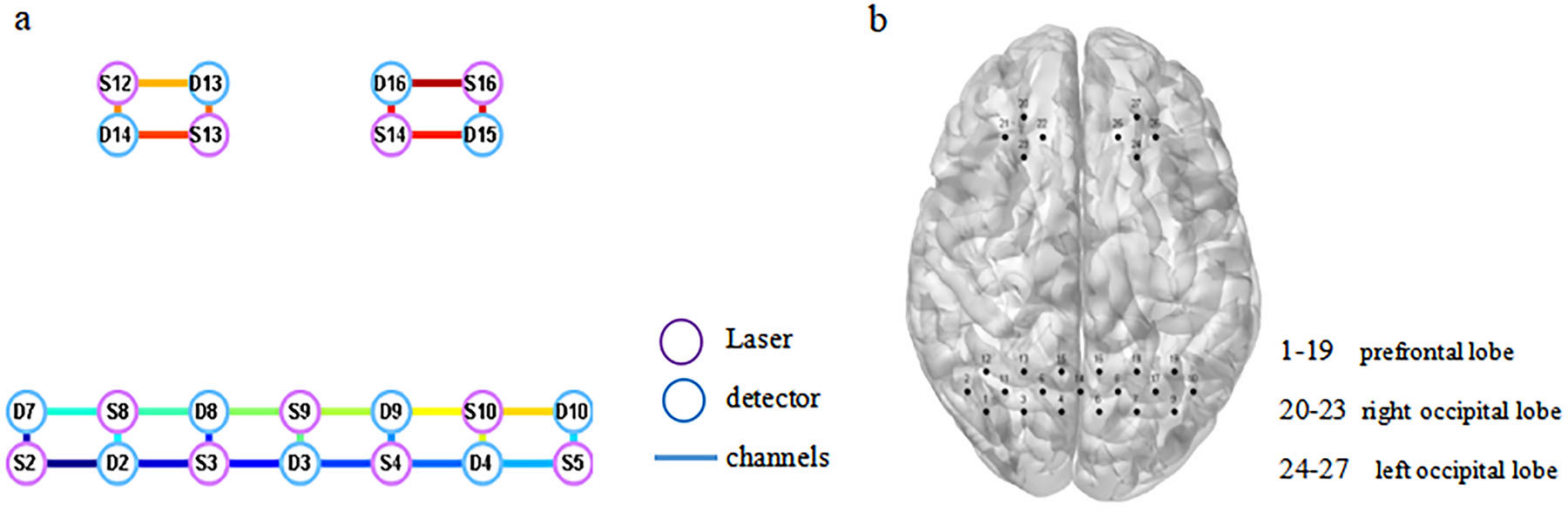
(a) Schematic illustration of the fNIRS probe layout; and (b) the topographical distribution of the fNIRS collecting channels.

The multi‐channel fNIRS system (NirScan; Danyang Huichuang Medical Equipment Co., Ltd., Jiangsu, China) was used to collect resting state brain function data of participants before and 3 weeks after rTMS intervention. The used wavelengths were 730, 808, and 850 nm. The sampling rate was 11 Hz. The fNIRS acquisition cap was designed based on the 10/20 international standard lead system, with a total of 11 laser sources, 11 light detectors, and 27 channels (Figure 2a). The head‐cap covered the subjects’ bilateral prefrontal lobe and occipital lobe, and a distance of 30 mm was maintained between the laser and the detector (Figure 2b).

In order to minimize the effect on the data, subjects were requested to relax for 10–15 min to regulate their breathing and mood before the start of the experiment. All subjects were requested to sit in a comfortable chair and avoid regular thinking and unnecessary movement. The duration of the fNIRS measurement was approximately 5 min.

The preprocessing module of the NirSpark software was used to preprocess the fNIRS signals. The initial setting for the signal standard deviation threshold was 6, while the peak threshold was set at 0.5. A method based on spline interpolation was adopted in this study to reduce movement artifacts. To eliminate the influence of heartbeat, breathing, and environmental noise, we applied a bandpass filtering with a range of 0.01 to 0.2 Hz to the detected raw optical signals. Subsequently, employing the modified Beer‐Lambert law, we converted the light signal into concentration data for ∆HbO2 and ∆HbR.

### 2.6 rsFC Analysis

**FIGURE 3:**
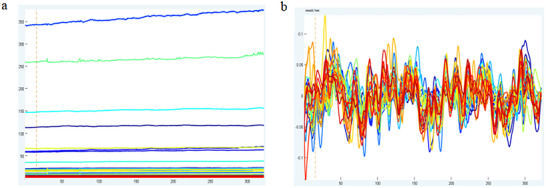
An example for the preprocessed result: (a) original signal; and (b) preprocessed signal.

The Network module of the NirSpark software was used to perform FC analysis. Previous studies have demonstrated that, in contrast to the ∆HbR signal, the ∆HbO2 signal possesses a better signal‐to‐noise ratio and is sensitive to regional cerebral blood oxygen alterations (Tang and Chan 2018). Therefore, we used only the ∆HbO2 data in this study. In the Network module, the resting‐state data of the subjects at each time point was extracted, and the ∆HbO2 content of each brain region in the time series was statistically analyzed. The Pearson correlation coefficient of ∆HbO2 in each channel and brain region within the time series was calculated, which is defined as the resting‐state FC strength (Figure 3).

### 2.7 Statistical Analysis

Statistical analyses were performed with SPSS V.25.0 software. Continuous variables with normal distribution were presented as means ± standard deviations (SD), while non‐normal distribution variables were reported as medians (interquartile range). The assumption of normality for continuous variables was tested using the Shapiro‐Wilks test. For normally distributed continuous variables, the two‐sample t‐test was used for comparison between groups, and the paired sample t‐test was used for within‐group comparisons. The chi‐squared test was used for categorical variables. The Mann‐Whitney U test was used to compare two groups in terms of non‐normal distribution variables, and the Wilcoxn signed‐rank test was used for within‐group comparisons. The NirSpark software was employed to carry out a t‐test on the average FC strength of the two groups of brain networks. A difference of *p* < 0.05 was considered statistically significant.

## 3 Results

### 3.1 Demographic and Clinical Characteristics

In total, 45 participants were evaluated, 3 of whom were excluded (two patients were discharged halfway through, and one patient did not cooperate in the re‐evaluation). Thus, 42 participants (*n* = 21 in each group) were finally included. There was no significant difference between the two groups in baseline characteristics like age, sex ratio, disease duration, years of education, type of stroke, and neuropsychological scale scores (*p* > 0.05). Details of the demographic data and corresponding tests are presented in Table 1.

**TABLE 1:** Demographics and neuropsychological data.

Variables	rTMS group (*N* = 21)	No‐stim control group (*N* = 21)	t/χ^2^/z value	*p* value
Age	59.76 ± 7.848	63.57 ± 9.485	−1.418	0.164
Gender (M/F)	16/5	17/4	—	0.500
Education (years)	9 (6, 15.5)	12 (7.5, 15)	−0.103	0.918
Disease duration (days)	48 (26.5, 98)	28 (25, 85)	−0.554	0.579
Type of stroke (hemorrhagic/ischemic)	12/9	16/5	1.714	0.190
MoCA scores	15.81 ± 4.622	15.71 ± 4.529	0.067	0.947
RAVLT scores	—	—	—	—
RAVLT‐IR	25.67 ± 7.151	25.62 ± 7.460	0.021	0.983
RAVLT‐DR	1 (0, 1.5)	0 (0, 1.0)	−1.231	0.225
ROCF scores	—	—	—	—
ROCF‐C	11 (7.5, 18)	9 (3, 17)	−0.982	0.325
ROCF‐DR	5 (1, 8)	5 (1.5, 5.5)	−0.483	0.639

Abbreviations: MoCA, montreal cognitive assessment; RAVLT, Rey auditory verbal learning test; RAVLT‐IR: RAVLT immediate recall; RAVLT‐DR, RAVLT delayed recall; ROCF, Rey‐osterrieth complex figure test; ROCF‐C, ROCF copying; ROCF‐DR, ROCF delayed recall.

### 3.2 Comparison of the Improvement of Neuropsychological Test in Two Groups

#### 3.2.1 RAVLT Score

**FIGURE 4:**
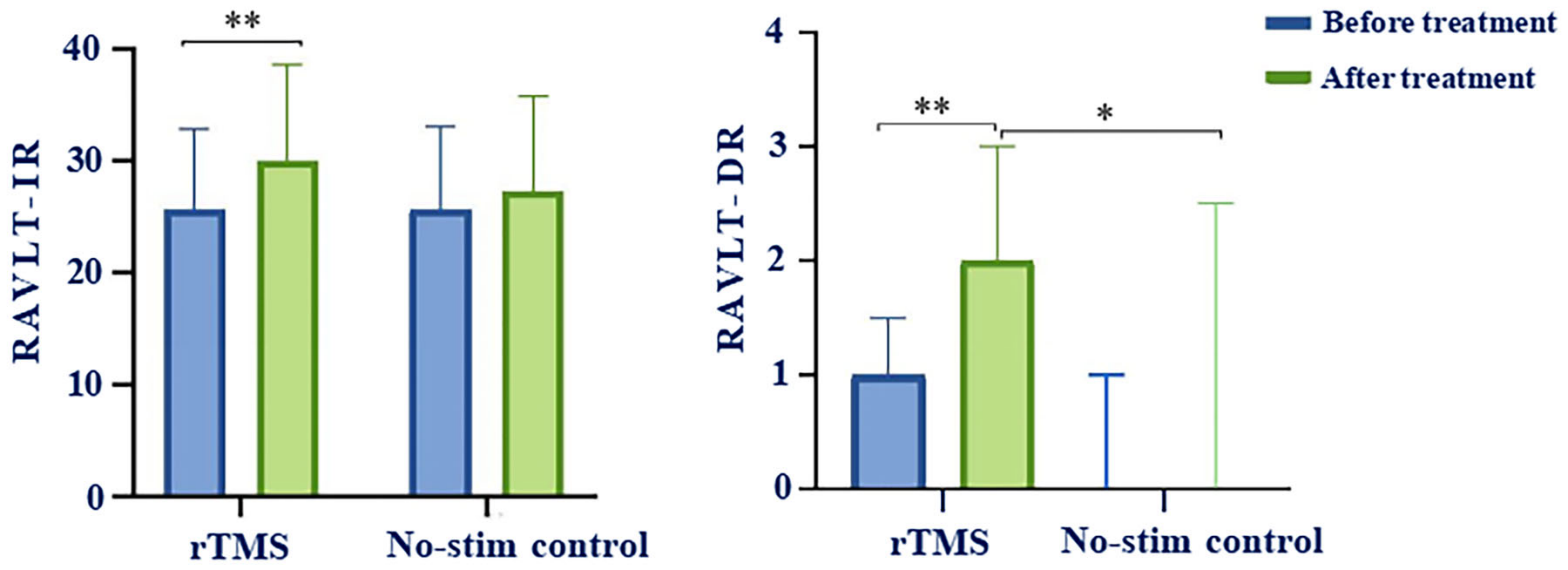
Comparison of the RAVLT‐IR and RAVLT‐DR scores in the rTMS group and the no‐stim control group, * *p* < 0.05, ** *p* < 0.01.

The results showed that the RAVLT‐IR and RAVLT‐DR scores of patients in the rTMS group were significantly higher than those before treatment (*p* < 0.01). In the no‐stim control group, the RAVLT‐IR and RAVLT‐DR scores were slightly improved compared to the baseline, with no statistically significant difference (Table 2 and Figure 4).

**TABLE 2:** Comparison of the improvement of RAVLT score between two groups.

Variables	rTMS group	No‐stim control group	t/z value	*p* value
RAVLT‐IR	29.95 ± 8.709**	27.33 ± 8.458	0.979	0.329
RAVLT‐DR	2 (1.5, 3.0)**	0 (0, 2.5)	−2.349	0.023

*Notes*: Compared with the same group before treatment, ** *p* < 0.01.

Abbreviations: RAVLT, Rey auditory verbal learning test; RAVLT‐IR: RAVLT immediate recall; RAVLT‐DR, RAVLT delayed recall.

Intergroup comparison revealed that the RAVLT‐DR score of the rTMS group was significantly improved compared to the no‐stim control group after treatment (*p* < 0.05), while there was no statistically significant disparity in the RAVLT‐IR score between the two groups (Table 2 and Figure 4).

#### 3.2.2 ROCF Score

**FIGURE 5:**
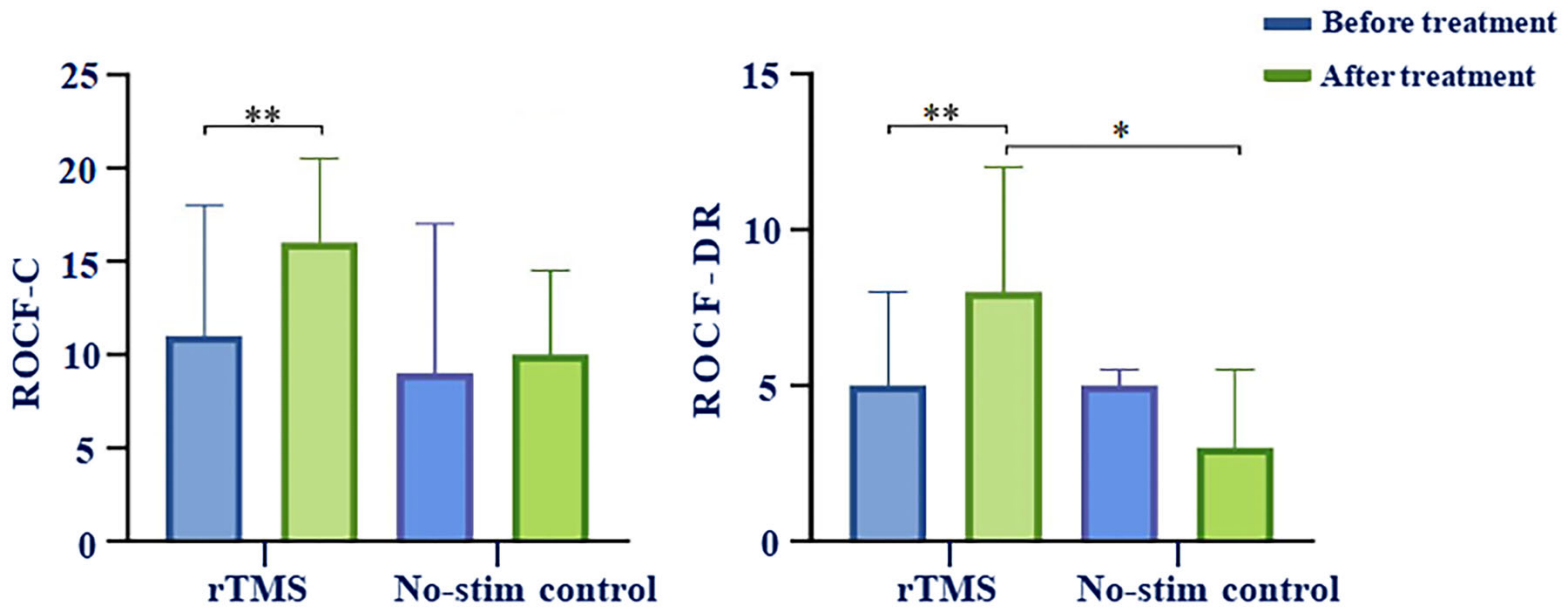
Comparison of the ROCF‐C and ROCF‐DR scores in the rTMS group and the no‐stim control group, * *p* < 0.05, ** *p* < 0.01.

The ROCF‐C and ROCF‐DR scores in the rTMS group were significantly enhanced compared to those before treatment (p < 0.05), while in the no‐stim control group were all improved after treatment but with no statistically significant difference (Table 3 and Figure 5).

**TABLE 3:** Comparison of the improvement of ROCF score between two groups.

Variables	rTMS group	No‐stim control group	z value	p value
ROCF‐C	16 (5, 20.5)*	10 (4.5, 14.5)	−1.791	0.074
ROCF‐DR	8 (3, 12)**	3 (2, 5.5)	−2.027	0.042

*Notes*: Compared with the same group before treatment, * p < 0.05, ** p < 0.01.

Abbreviations: ROCF, Rey‐Osterrieth Complex Figure Test; ROCF‐C, ROCF copying; ROCF‐DR, ROCF delayed recall.

Intergroup comparison revealed that the ROCF‐DR score of the rTMS group was significantly improved compared to the no‐stim control group after treatment (*p* < 0.05), while there was no statistically significant disparity in the ROCF‐C score between the two groups (Table 3 and Figure 5).

#### 3.2.3 MoCA Score

**FIGURE 6:**
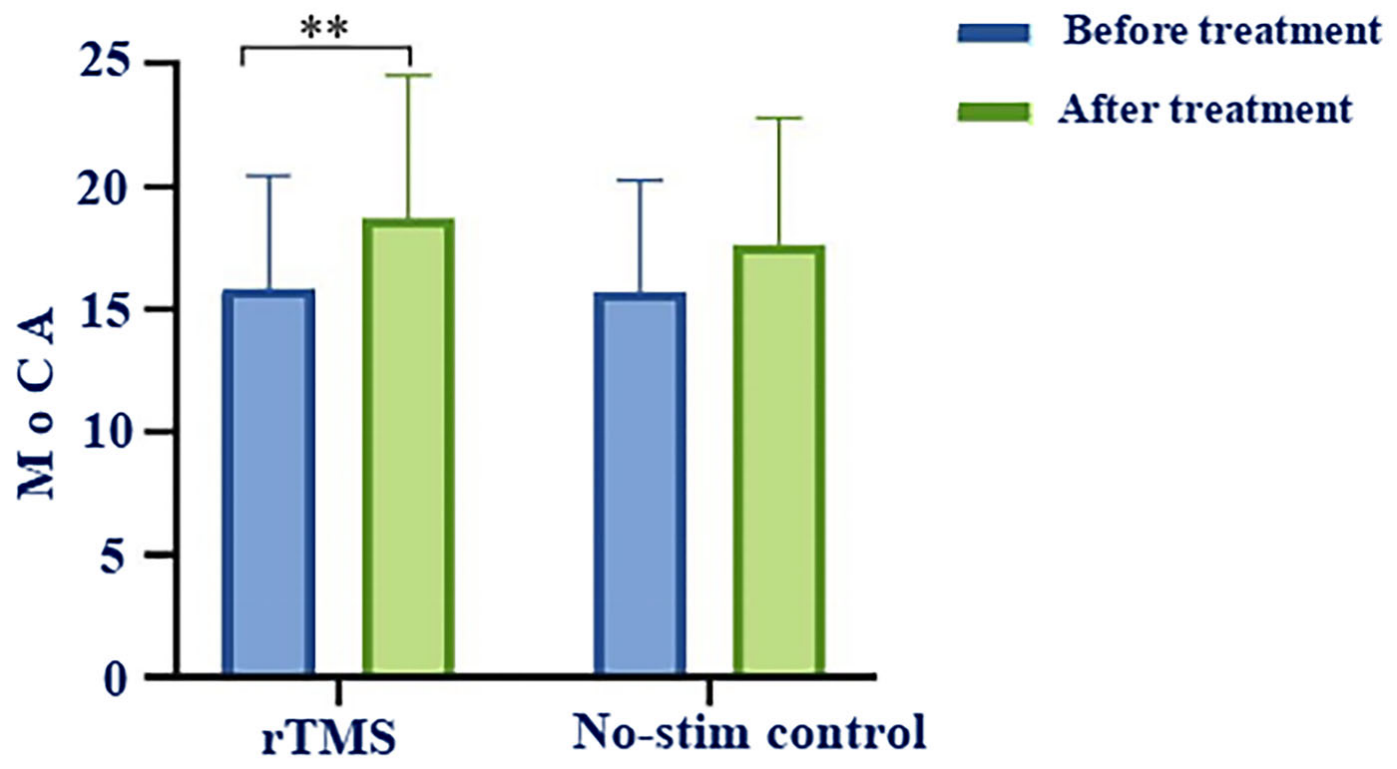
Comparison of the MoCA scores in the rTMS group and the no‐stim control group, ** *p* < 0.01.

After 3 weeks of treatment, the MOCA score in the rTMS group increased significantly (*p* < 0.01). The MOCA score in the no‐stim control group was marginally higher than that before treatment, yet there was no statistically significant difference (Table 4 and Figure 6).

**TABLE 4:** Comparison of the improvement of MoCA score between two groups.

Variables	rTMS group	No‐stim control group	*t* value	*p* value
MoCA	18.67 ± 5.868**	17.62 ± 5.162	0.614	0.543

*Note*: Compared with the same group before treatment, ** *p* < 0.01.

Abbreviations: MoCA, montreal cognitive assessment.

The improvement of the MoCA score for rTMS group was higher than that in the no‐stim control group after 3 weeks treatment. However, there was no statistically significant disparity (*p* > 0.05). See in Table 4 and Figure 6.

### 3.3 rsFC

**FIGURE 7:**
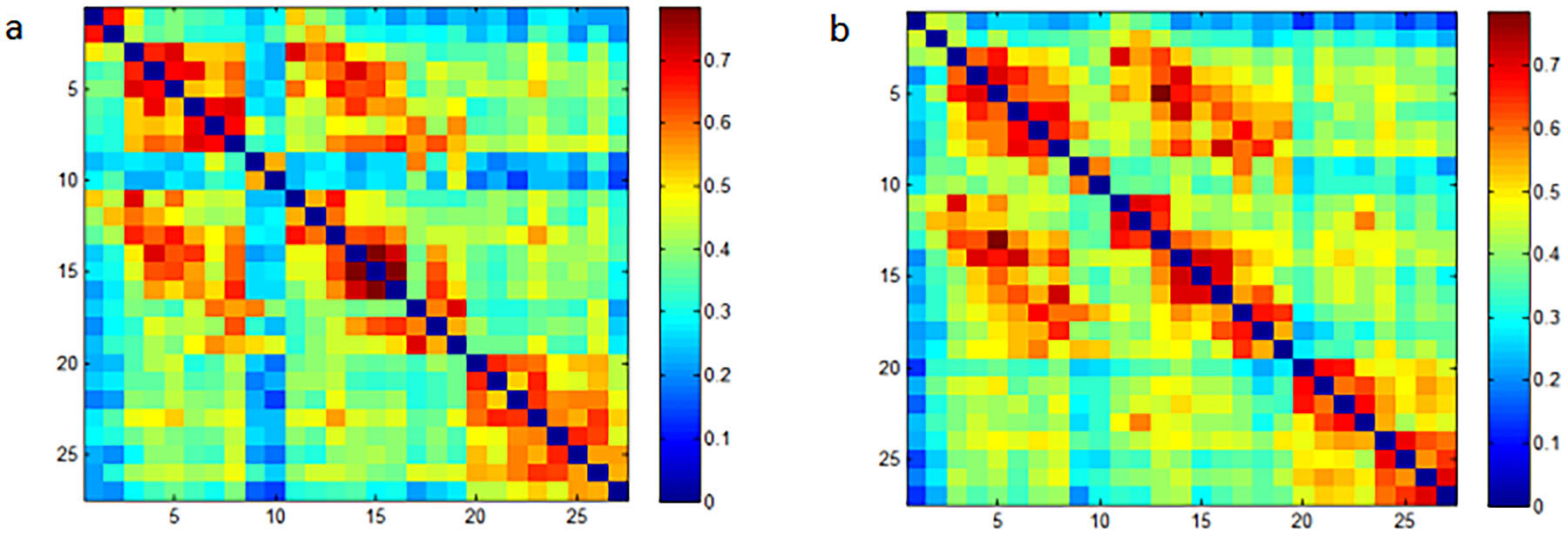
The rsFC matrices for the rTMS group: (a) before treatment; and (b) after treatment.

**FIGURE 8:**
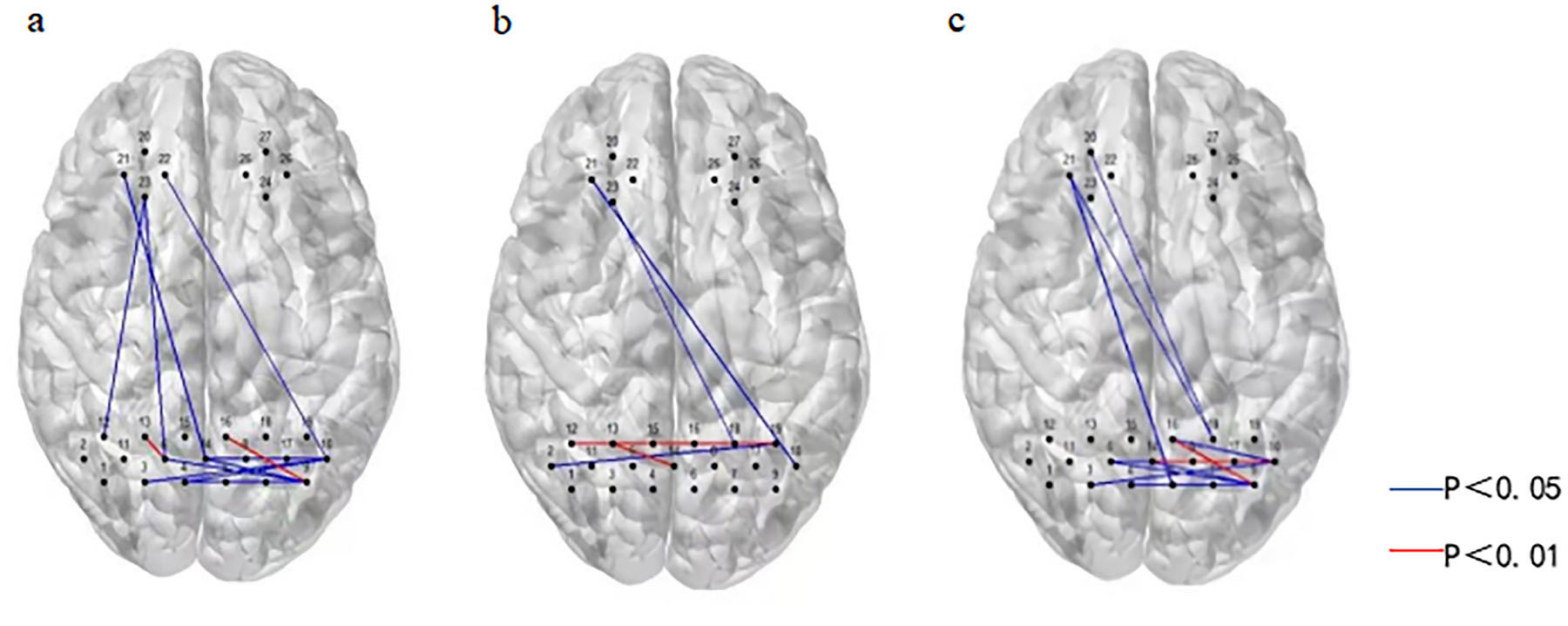
Outcomes of differential rsFC in two groups after treatment: (a) intragroup comparison within the rTMS group; (b) intragroup comparison within the no‐stim control group; (c) comparison between the two groups after treatment.

**FIGURE 9:**
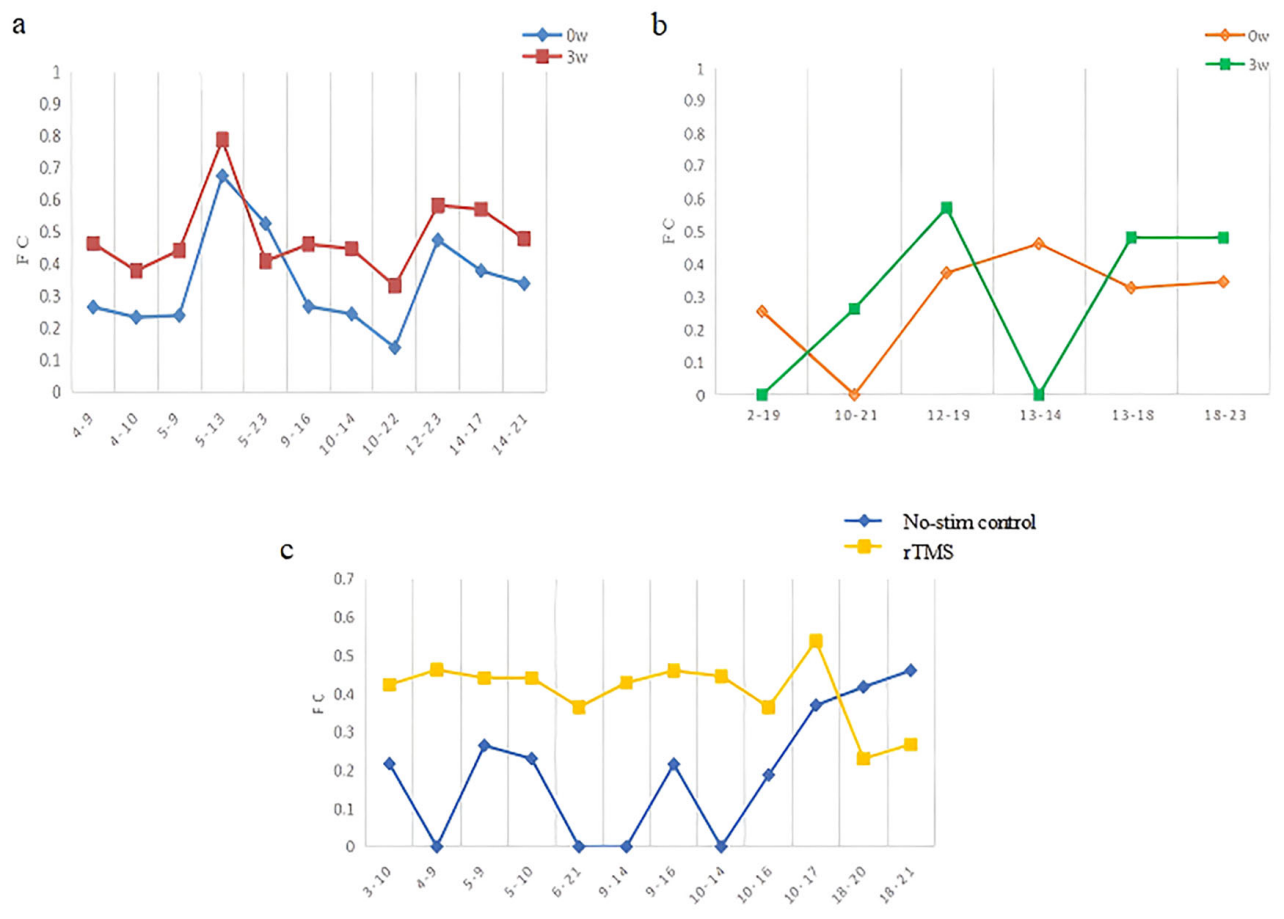
The rsFC values of the differential channels before and after treatment in two groups: (a) intragroup comparison within the rTMS group; (b) intragroup comparison within the no‐stim control group; and (c) comparison between the two groups after treatment.

Figure 7 shows the rsFC matrices for the rTMS group before and after treatment. Red and blue colors represent positive and negative rsFC, respectively. The results show that the strength of the average rsFC was enhanced after treatment. Specifically, the FC within the prefrontal lobe and between the bilateral prefrontal lobe and the right occipital lobe was significantly enhanced after treatment (*p* < 0.05, uncorrected, as shown in Figure 8a and Figure 9a). Figure 8b and Figure 9b show that there are only a few irregular and statistically significant rsFC between the left prefrontal lobe and the right occipital lobe as well as within the prefrontal lobe in the no‐stim control group after treatment (*p* < 0.05, uncorrected). Compared with controls, the rTMS group demonstrated significantly enhanced rsFC within the prefrontal lobe and between the left prefrontal lobe and right occipital lobe after treatment (*p* < 0.05, uncorrected, as shown in Figure 8c and Figure 9c).

## 4 Discussion

This study reported the effects of high‐frequency rTMS at the left parietal lobe on the improvement of memory function in post‐stroke patients. Our results revealed that the RAVLT‐DR and ROCF‐DR scores improved significantly more in the rTMS group after treatment in contrast to the controls. These changes accompanied altered activation of local frontal regions and enhanced rsFC within the frontal network and between the left prefrontal lobe and the right occipital lobe. These results indicated that the rTMS group attained better neural function recovery, and high‐frequency rTMS on the left parietal lobe was effective in ameliorating memory impairment after stroke.

Some studies showed that high‐frequency rTMS therapy could conspicuously enhance the overall cognitive function of stroke patients. This might be associated with the excitation of the cortex, the reopening of the frontal‐striatal loop, the facilitation of regional reorganization of cortical function, and the augmentation of hippocampal synaptic plasticity and so on (Hulst et al. 2017; Soundara Rajan et al. 2017, Burke et al. 2019; Hong et al. 2021; Li and Xiao 2024). In the rTMS therapy for cognitive impairment, the most common target is the dorsolateral prefrontal cortex. However, an increasing number of neuroimaging studies have indicated that other brain regions are involved in the task of memory, including the medial temporal lobe, parietal lobe, and the precuneus, etc. (Cavanna and Trimble 2006; Manenti et al. 2012; Lech and Suchan 2013). One area that is gaining escalating attention in memory research is the parietal cortex, which mainly participates in the processing of information concerning somatosensory, visual, and spatial cognition, working memory, episodic memory, and attention (Sack AT 2009; Myskiw and Izquierdo 2012; Berlucchi and Vallar 2018). A study has revealed that the parietal lobe genuinely contributes to situation memory beyond the attention process (Hutchinson et al. 2014). Bjekić et al. (Bjekić et al. 2019) found that transcranial direct current stimulation over the posterior parietal cortex of healthy individuals can improve associative memory performance. Follow‐up studies revealed that high‐frequency rTMS of the left lateral parietal lobe significantly enhanced the functional connectivity within the hippocampal network and ameliorated declarative memory performance and the accuracy of spatial memory tasks in healthy subjects (Wang et al. 2014; Nilakantan et al. 2017). Cotelli et al. (Cotelli et al. 2012) performed 20 Hz rTMS stimulation in the left inferior parietal lobules of an individual with amnestic mild cognitive impairment, and observed an improvement on the delayed recall of the auditory‐verbal learning test and the primacy effect of serial position curve task. In this study, the MoCA scores, RAVLT, and ROCF‐DR scores of patients with post‐stroke memory impairment were notably improved after high‐frequency rTMS of the left parietal lobe, which was in line with the above studies, suggesting that stimulating the left parietal lobe of right‐handed individuals can enhance episodic memory and visual‐spatial memory function, further substantiating the role of the parietal lobe in the memory process.

Our further analysis with fNIRS revealed that the rsFC between the left prefrontal lobe and the right occipital lobe and within the prefrontal lobe was significantly enhanced after rTMS treatment. This indicated that the efficiency of information exchange among different parts of the cortex declined in patients with post‐stroke memory impairment. After rTMS stimulation of the left parietal lobe, it had a distant influence by regulating neural activity, neurotransmitter release, and cerebral blood flow, thereby strengthening the functional connections of the prefrontal cortex and the occipital lobe to improve memory function. We mainly focused on the downstream network effects of left parietal rTMS. Firstly, our fNIRS probe coverage was limited to the prefrontal and occipital regions due to the head‐cap type and patient compliance. Therefore, the direct hemodynamic effects of the left parietal stimulation were not captured. Secondarily, rTMS could modulate large‐scale brain networks, including downstream of the stimulation site, by propagating pulses across functionally connected systems (Battelli et al. 2017). Garcia et al. found that rTMS stimulation of the left intraparietal sulcus caused significant changes in neural activity in the lateral prefrontal cortex (downstream region), but did not substantially impact the allegiance of nodes directly under the stimulation site, confirming the presence of remote downstream network modulation (Garcia et al. 2020). In addition, the realization of neural function depends on the cooperation of multiple brain regions. The prefrontal cortex (particularly the dorsolateral prefrontal lobe), which is an important area for memory in the brain (Miller 2000), plays a crucial role in the encoding and retrieval of memory. The right dorsolateral prefrontal lobe is associated with episodic memory (Wagner et al. 1998), and its damage can result in a severe deterioration of self‐memory. Previous studies have reported that visual spatial memory is the outcome of the synergetic action of the parietal lobe and the prefrontal cortex (Suchan et al. 2006; Sahu and Tseng 2021). The occipital lobe is situated posterior to the parietal lobe and functions as the visual cortical center. During the processing of visual information, it connects visual, auditory, linguistic, and other executive functions (Le et al. 2017). Visual information is inputted from the lateral geniculate nucleus in the thalamus and then outputted through the primary visual cortex via the dorsal and ventral streams. The ventral stream connects the lateral temporal lobe and the occipital‐temporal region, being responsible for processing recognition information such as face, object, and scene recognition (Gilbert and Li 2013). The dorsal stream projects from the primary visual cortex through the parietal region to the prefrontal cortex, precentral gyrus, and the medial temporal lobe, participating in spatial working memory, visual‐guided behavior, and spatial position information processing, and helping to construct spatial features (Jacobs et al. 2015; Gallivan and Goodale 2018). Besides the anatomical connections, imaginging studies has further affirmed the close functional bond in the two circuits (Yeatman et al. 2014). Bu et al. (Bu et al. 2019) investigated the alterative FC of patients with amnestic mild cognitive impairment mainly occurred between the bilateral prefrontal lobe and occipital lobe. Zhang et al. (Zhang et al. 2024) found that the FC from the prefrontal lobe to the occipital lobe and from the prefrontal lobe to the parietal lobe were significantly reduced in patients with mild cognitive impairment. Our findings are analogous to the aforesaid results, indicating that the prefrontal lobe and the occipital lobe constitute one of the key impaired cortices in memory disorders.

The theory of brain plasticity and the reorganization and compensation of central nervous system neurons serve as an important theoretical foundation for rehabilitation treatment. Reorganizing the activity of the existing cortical/subcortical networks can significantly facilitate the improvement of post‐stroke functional impairment (Carter et al. 2012). We presume that high‐frequency rTMS at the left parietal lobe enhances its information processing capabilities, and then, through neural conduction circuits inducing the activation of the prefrontal and occipital cortical networks, resulting in the enhancement of intracortical and intercortical functional connectivity and enhancing the integration effect of cognitive information to improve memory function and increase the efficiency of the memory system. This might be one of the mechanisms through which rTMS ameliorates memory impairment in patients with stroke during the recovery period.

There were no statistically significant between‐group differences in post‐treatment scores for RAVLT‐IR and the MoCA total score, and we considered the following possible reasons. First, the sample size was relatively small, resulting in limited statistical power to detect small but clinically meaningful differences between groups in RAVLT‐IR and the MoCA total score. Second, our study included patients with different stroke lesion locations. Although there were no statistically significant differences in age, gender, education level, disease duration, and other factors between the two groups, there may still be potential differences in neuroplasticity, which may affect patients' response to rTMS treatment, resulting in no significant between‐group differences in changes in some dimensions of the scale. Subgroup analyses may be more useful to reveal the characteristics of the population that may have a greater benefit from treatment.

## 5 Limitations of the Study

First, the sample size was relatively small, and the proportion of female patients was lower, which might influence the generalizability of our current research findings. Second, coil placement using the international 10–20 system may be influenced by individual anatomical variability or operator subjectivity. Third, employing resting‐state FC solely is incapable of fully reflecting the alterations in brain function. Future studies should further enlarge the sample size, employ neuronavigation for more precise and individualized targeting, and combine multiple brain function evaluation indicators to further investigate the effects of changes in different functional networks on the recovery of memory impairment, which will assist in further clarifying the neural mechanisms by which rTMS improves memory impairment.

## 6 Conclusions

In conclusion, our results suggest that supplementing conventional treatment with high‐frequency rTMS to the left parietal lobe can improve memory function for patients with post‐stroke memory impairment, with accompanying increased FC between the left prefrontal lobe and the right occipital lobe and within the prefrontal lobe. This might be one of the mechanisms through which rTMS ameliorates memory impairment in patients during the post‐stroke recovery period.

## Author Contributions

**Luhui Cai**: Writing – original draft, formal analysis, review. **Yuye Sun**: Data curation, validation. **Yijiang Li**: Software. **Peirong Wu**: Visualization. **Mingdong Wei**: Experiments. **Yinuo Bi**: Experiments. **Chaowen Wang**: Experiments. **Wenyu Jiang**: Project administration. All authors contributed to editorial changes in the manuscript. All authors read and approved the final manuscript. All authors have participated sufficiently in the work and agreed to be accountable for all aspects of the work.

## Funding

This work was supported by the grant from the Natural Science Foundation of Guangxi Zhuang Autonomous Region (Grant No. 2016GXNSFAA38011 and 2023JJA140607). We thank the Key Cultivation Discipline of Neurology in Guangxi Zhuang Autonomous Region and the National Key Research and Development Program (2018YFC2001700) for their support.

## Ethics Statement

This study was approved by the Jiangbin Hospital of Guangxi Zhuang Autonomous Region Ethics Committee (ID: KY‐YJS‐2020‐02). All procedures were carried out in accordance with relevant guidelines and regulations.

## Consent

All authors read and approved the final manuscript. All authors have participated sufficiently in the work and agreed to be accountable for all aspects of the work.

## Conflicts of Interest

The authors declare no comflicts of interest.

## Data Availability

The datasets generated and/or analysed during the current study are not publicly available due to the policies of our laboratory and the confidentiality agreements signed with the patients but are available from the corresponding author on reasonable request.
